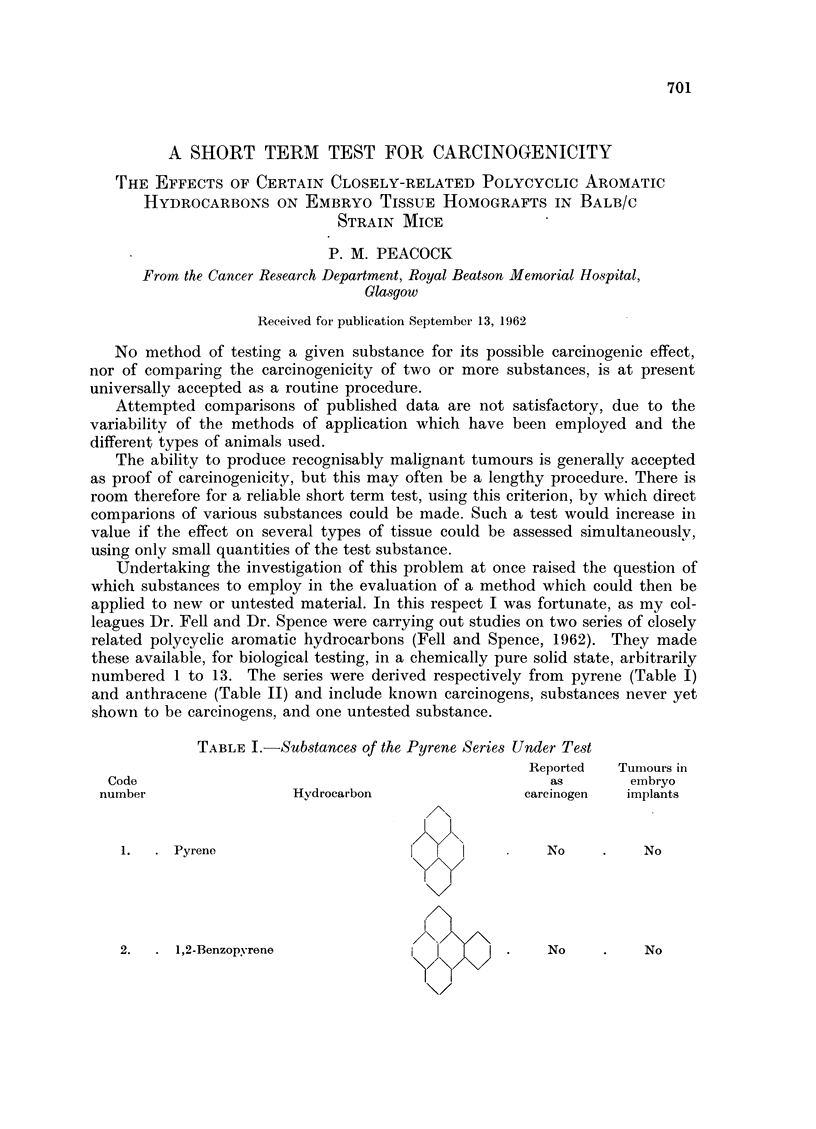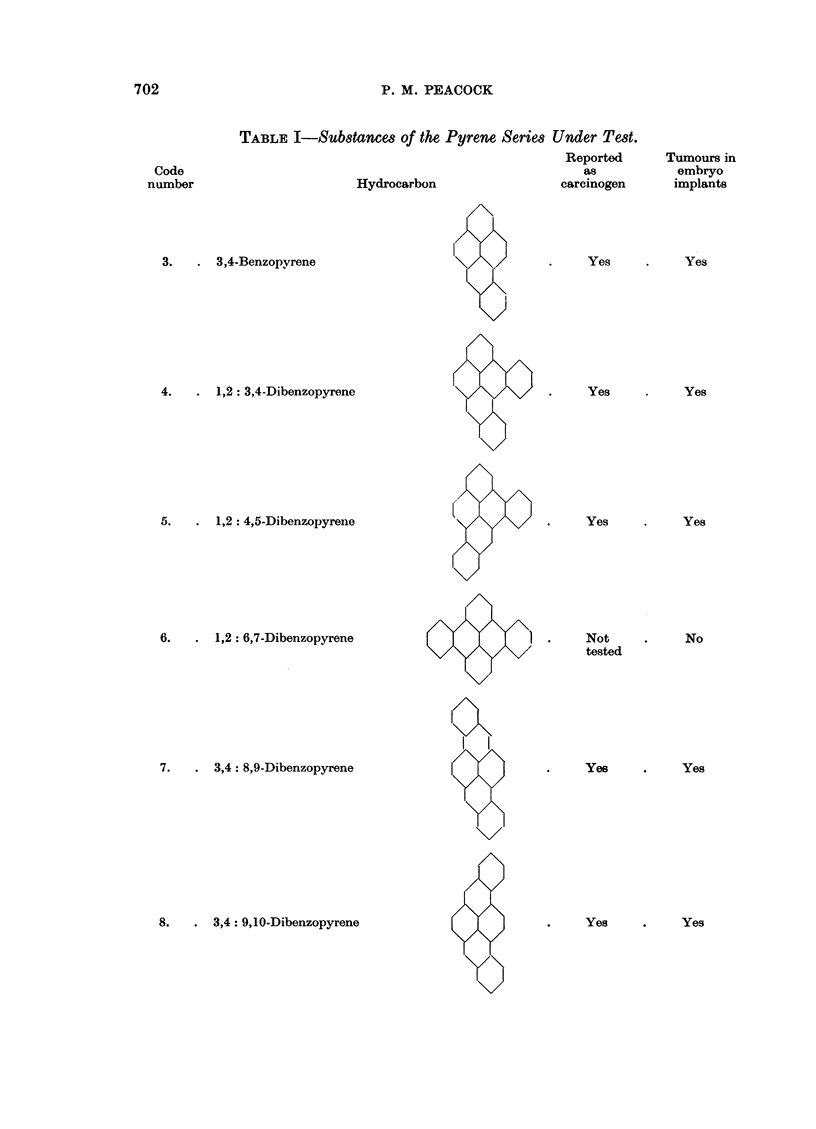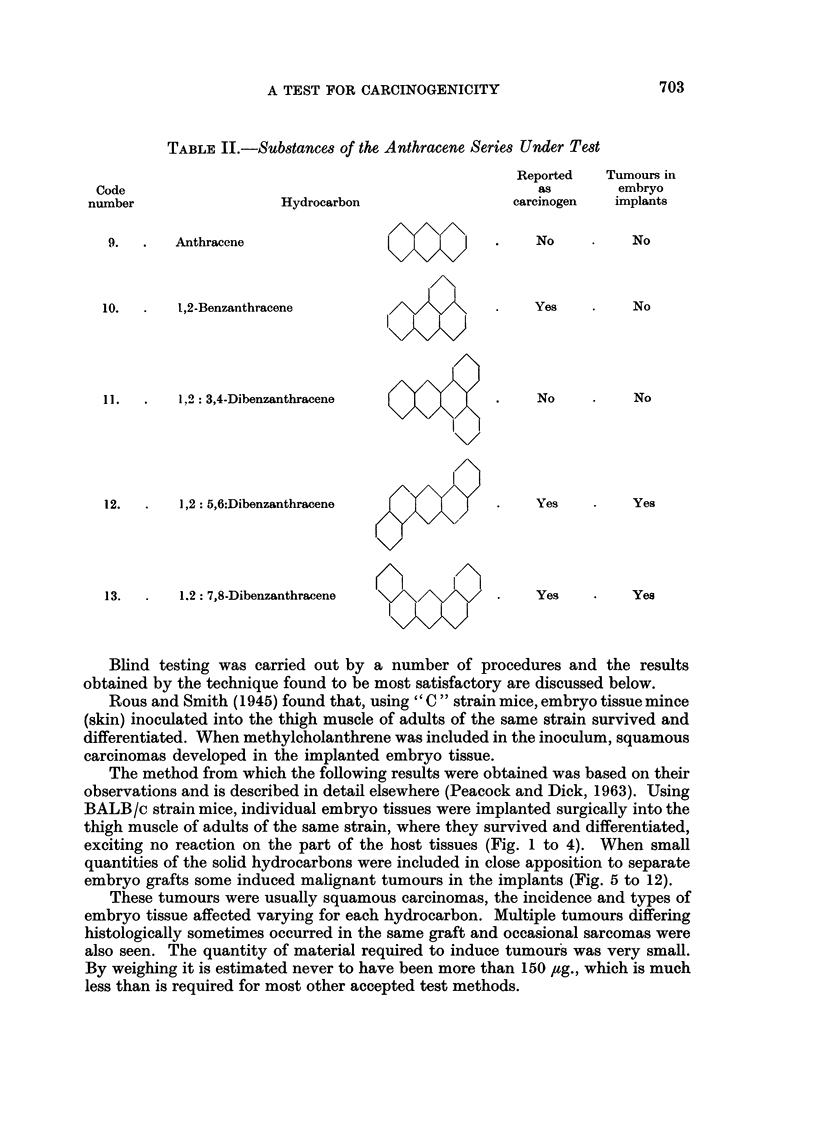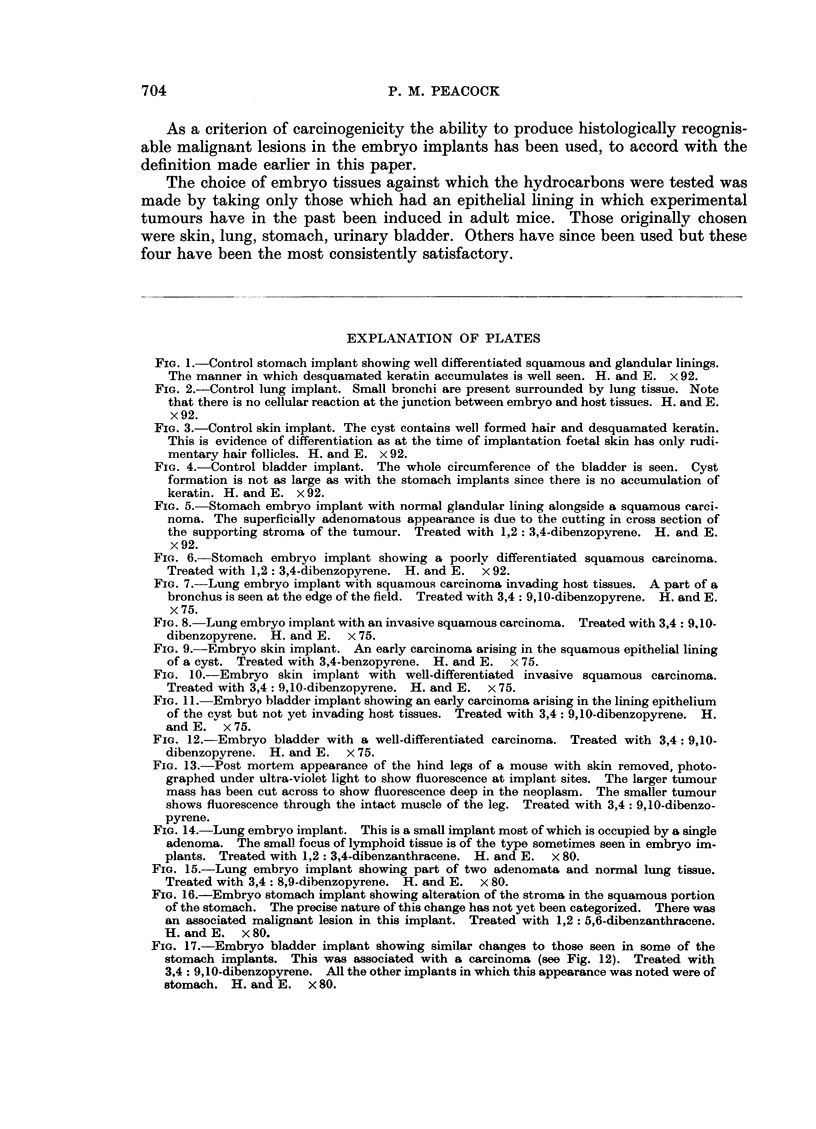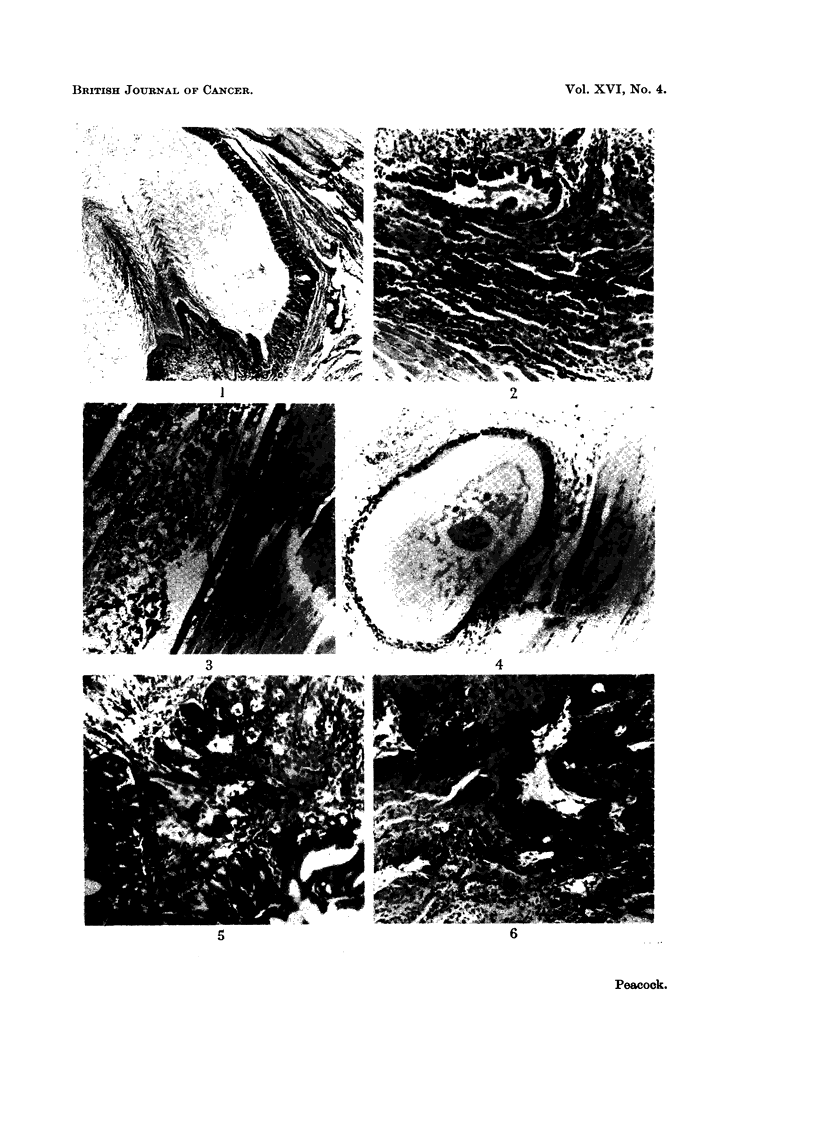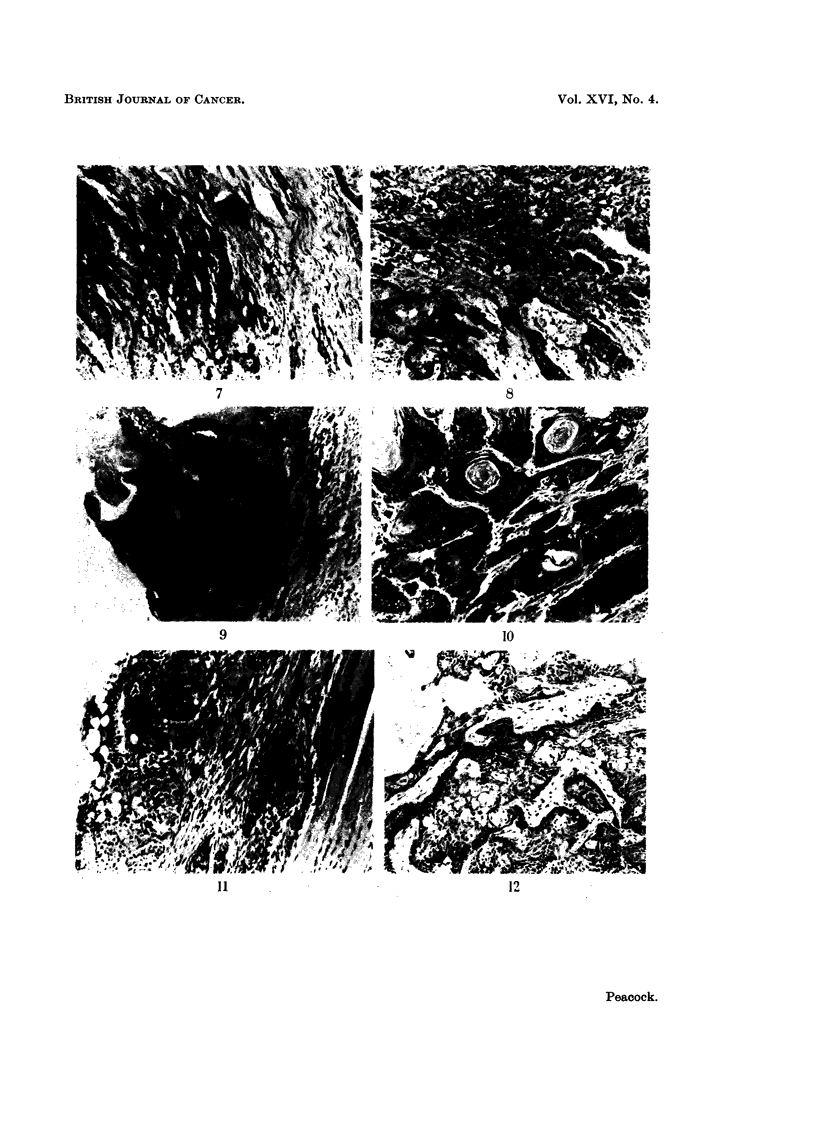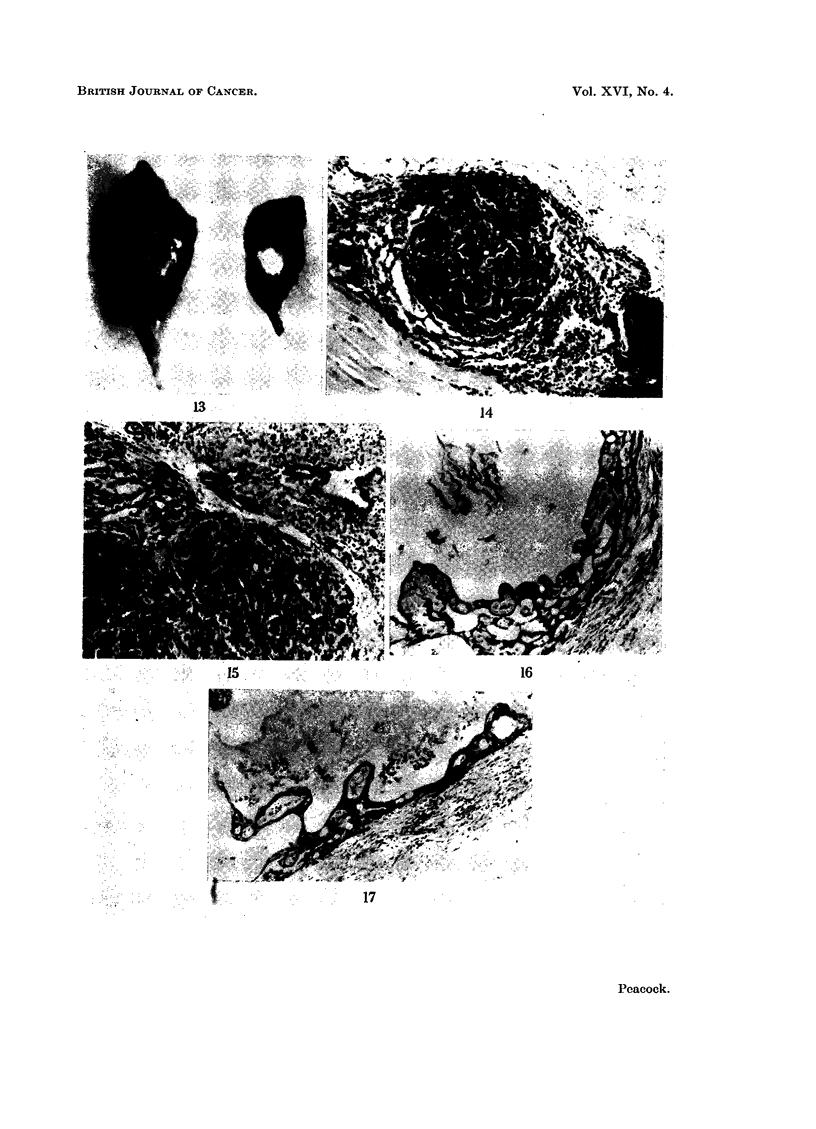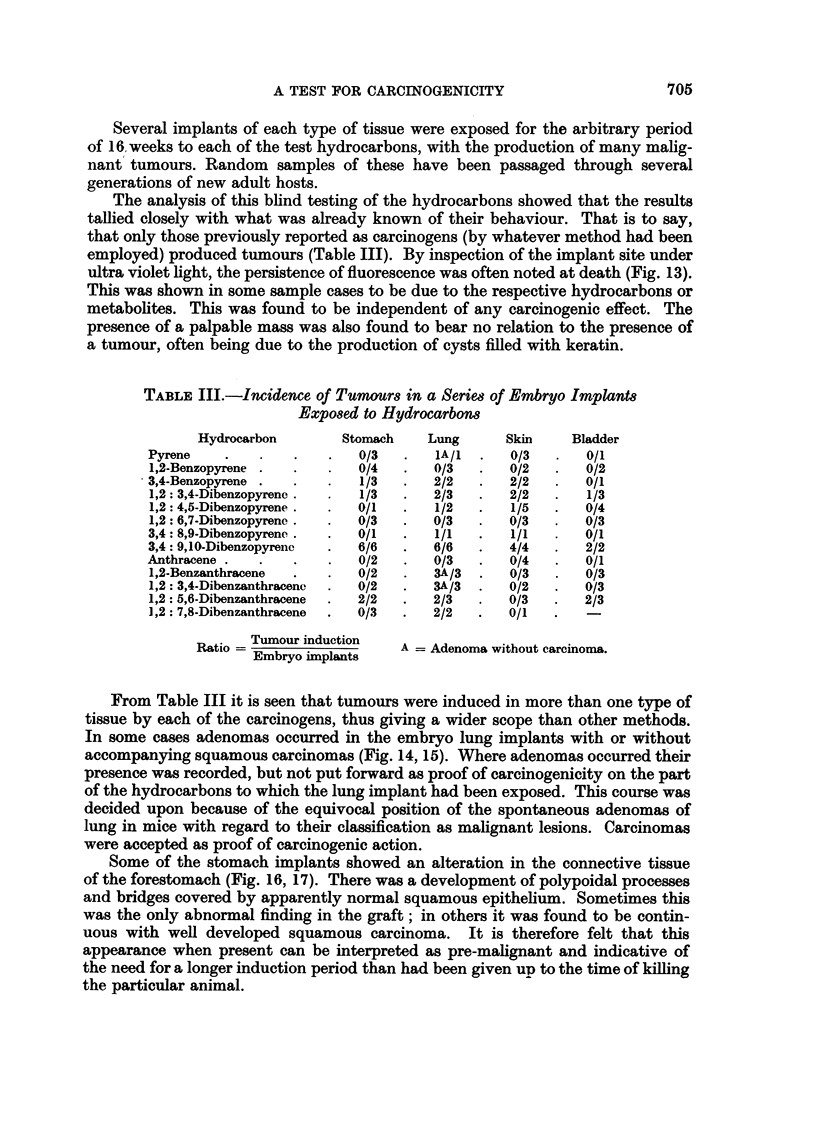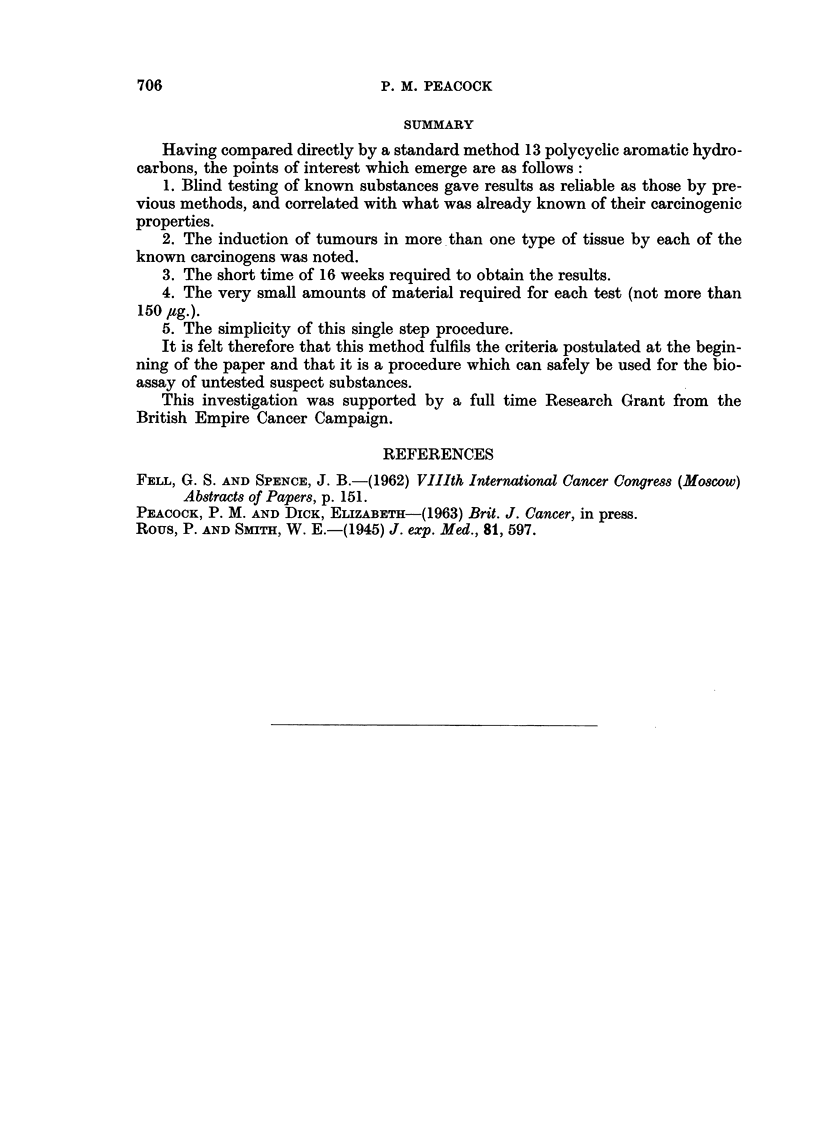# A Short Term Test for Carcinogenicity

**DOI:** 10.1038/bjc.1962.81

**Published:** 1962-12

**Authors:** P. M. Peacock

## Abstract

**Images:**


### A SHORT TERM TEST FOR CARCINOGENICITY

### THE EFFECTS OF CERTAIN CLOSELY-RELATED POLYCYCLIC AROMATIC HYDROCARBONS ON EMBRYO TISSUE HOMOGRAFTS IN BALB/C STRAIN MICE

P. M. PEACOCK

*From the Cancer Research Department, Royal Beatson Memorial Hospital, Glasgow*

Received for publication September 13, 1962

No method of testing a given substance for its possible carcinogenic effect, nor of comparing the carcinogenicity of two or more substances, is at present universally accepted as a routine procedure.

Attempted comparisons of published data are not satisfactory, due to the variability of the methods of application which have been employed and the different types of animals used.

The ability to produce recognisably malignant tumours is generally accepted as proof of carcinogenicity, but this may often be a lengthy procedure. There is room therefore for a reliable short term test, using this criterion, by which direct comparions of various substances could be made. Such a test would increase in value if the effect on several types of tissue could be assessed simultaneously, using only small quantities of the test substance.

Undertaking the investigation of this problem at once raised the question of which substances to employ in the evaluation of a method which could then be applied to new or untested material. In this respect I was fortunate, as my colleagues Dr. Fell and Dr. Spence were carrying out studies on two series of closely related polycyclic aromatic hydrocarbons (Fell and Spence, 1962). They made these available, for biological testing, in a chemically pure solid state, arbitrarily numbered 1 to 13. The series were derived respectively from pyrene (Table I) and anthracene (Table II) and include known carcinogens, substances never yet shown to be carcinogens, and one untested substance.

TABLE I.—*Substances of the Pyrene Series Under Test*

| Code number | Hydrocarbon | | Reported as carcinogen | Tumours in embryo implants |
|---|---|---|---|---|
| 1. | Pyrene |  | No | No |
| 2. | 1,2-Benzopyrene |  | No | No |

TABLE I—*Substances of the Pyrene Series Under Test.*

| Code number | Hydrocarbon | | Reported as carcinogen | Tumours in embryo implants |
|---|---|---|---|---|
| 3. | 3,4-Benzopyrene |  | Yes | Yes |
| 4. | 1,2 : 3,4-Dibenzopyrene |  | Yes | Yes |
| 5. | 1,2 : 4,5-Dibenzopyrene |  | Yes | Yes |
| 6. | 1,2 : 6,7-Dibenzopyrene |  | Not tested | No |
| 7. | 3,4 : 8,9-Dibenzopyrene |  | Yes | Yes |
| 8. | 3,4 : 9,10-Dibenzopyrene |  | Yes | Yes |

TABLE II.—*Substances of the Anthracene Series Under Test*

| Code number | Hydrocarbon | | Reported as carcinogen | Tumours in embryo implants |
|---|---|---|---|---|
| 9. | Anthracene |  | No | No |
| 10. | 1,2-Benzanthracene |  | Yes | No |
| 11. | 1,2 : 3,4-Dibenzanthracene |  | No | No |
| 12. | 1,2 : 5,6:Dibenzanthracene |  | Yes | Yes |
| 13. | 1,2 : 7,8-Dibenzanthracene |  | Yes | Yes |

Blind testing was carried out by a number of procedures and the results obtained by the technique found to be most satisfactory are discussed below.

Rous and Smith (1945) found that, using "C" strain mice, embryo tissue mince (skin) inoculated into the thigh muscle of adults of the same strain survived and differentiated. When methylcholanthrene was included in the inoculum, squamous carcinomas developed in the implanted embryo tissue.

The method from which the following results were obtained was based on their observations and is described in detail elsewhere (Peacock and Dick, 1963). Using BALB/c strain mice, individual embryo tissues were implanted surgically into the thigh muscle of adults of the same strain, where they survived and differentiated, exciting no reaction on the part of the host tissues (Fig. 1 to 4). When small quantities of the solid hydrocarbons were included in close apposition to separate embryo grafts some induced malignant tumours in the implants (Fig. 5 to 12).

These tumours were usually squamous carcinomas, the incidence and types of embryo tissue affected varying for each hydrocarbon. Multiple tumours differing histologically sometimes occurred in the same graft and occasional sarcomas were also seen. The quantity of material required to induce tumours was very small. By weighing it is estimated never to have been more than 150 $\mu$g., which is much less than is required for most other accepted test methods.

As a criterion of carcinogenicity the ability to produce histologically recognisable malignant lesions in the embryo implants has been used, to accord with the definition made earlier in this paper.

The choice of embryo tissues against which the hydrocarbons were tested was made by taking only those which had an epithelial lining in which experimental tumours have in the past been induced in adult mice. Those originally chosen were skin, lung, stomach, urinary bladder. Others have since been used but these four have been the most consistently satisfactory.

---

### EXPLANATION OF PLATES

FIG. 1.—Control stomach implant showing well differentiated squamous and glandular linings. The manner in which desquamated keratin accumulates is well seen. H. and E. ×92.

FIG. 2.—Control lung implant. Small bronchi are present surrounded by lung tissue. Note that there is no cellular reaction at the junction between embryo and host tissues. H. and E. ×92.

FIG. 3.—Control skin implant. The cyst contains well formed hair and desquamated keratin. This is evidence of differentiation as at the time of implantation foetal skin has only rudimentary hair follicles. H. and E. ×92.

FIG. 4.—Control bladder implant. The whole circumference of the bladder is seen. Cyst formation is not as large as with the stomach implants since there is no accumulation of keratin. H. and E. ×92.

FIG. 5.—Stomach embryo implant with normal glandular lining alongside a squamous carcinoma. The superficially adenomatous appearance is due to the cutting in cross section of the supporting stroma of the tumour. Treated with 1,2 : 3,4-dibenzopyrene. H. and E. ×92.

FIG. 6.—Stomach embryo implant showing a poorly differentiated squamous carcinoma. Treated with 1,2 : 3,4-dibenzopyrene. H. and E. ×92.

FIG. 7.—Lung embryo implant with squamous carcinoma invading host tissues. A part of a bronchus is seen at the edge of the field. Treated with 3,4 : 9,10-dibenzopyrene. H. and E. ×75.

FIG. 8.—Lung embryo implant with an invasive squamous carcinoma. Treated with 3,4 : 9,10-dibenzopyrene. H. and E. ×75.

FIG. 9.—Embryo skin implant. An early carcinoma arising in the squamous epithelial lining of a cyst. Treated with 3,4-benzopyrene. H. and E. ×75.

FIG. 10.—Embryo skin implant with well-differentiated invasive squamous carcinoma. Treated with 3,4 : 9,10-dibenzopyrene. H. and E. ×75.

FIG. 11.—Embryo bladder implant showing an early carcinoma arising in the lining epithelium of the cyst but not yet invading host tissues. Treated with 3,4 : 9,10-dibenzopyrene. H. and E. ×75.

FIG. 12.—Embryo bladder with a well-differentiated carcinoma. Treated with 3,4 : 9,10-dibenzopyrene. H. and E. ×75.

FIG. 13.—Post mortem appearance of the hind legs of a mouse with skin removed, photographed under ultra-violet light to show fluorescence at implant sites. The larger tumour mass has been cut across to show fluorescence deep in the neoplasm. The smaller tumour shows fluorescence through the intact muscle of the leg. Treated with 3,4 : 9,10-dibenzopyrene.

FIG. 14.—Lung embryo implant. This is a small implant most of which is occupied by a single adenoma. The small focus of lymphoid tissue is of the type sometimes seen in embryo implants. Treated with 1,2 : 3,4-dibenzanthracene. H. and E. ×80.

FIG. 15.—Lung embryo implant showing part of two adenomata and normal lung tissue. Treated with 3,4 : 8,9-dibenzopyrene. H. and E. ×80.

FIG. 16.—Embryo stomach implant showing alteration of the stroma in the squamous portion of the stomach. The precise nature of this change has not yet been categorized. There was an associated malignant lesion in this implant. Treated with 1,2 : 5,6-dibenzanthracene. H. and E. ×80.

FIG. 17.—Embryo bladder implant showing similar changes to those seen in some of the stomach implants. This was associated with a carcinoma (see Fig. 12). Treated with 3,4 : 9,10-dibenzopyrene. All the other implants in which this appearance was noted were of stomach. H. and E. ×80.

1

2

3

4

5

6

Peacock.

7

8

9

10

11

12

13

14

15

16

17

Several implants of each type of tissue were exposed for the arbitrary period of 16 weeks to each of the test hydrocarbons, with the production of many malignant tumours. Random samples of these have been passaged through several generations of new adult hosts.

The analysis of this blind testing of the hydrocarbons showed that the results tallied closely with what was already known of their behaviour. That is to say, that only those previously reported as carcinogens (by whatever method had been employed) produced tumours (Table III). By inspection of the implant site under ultra violet light, the persistence of fluorescence was often noted at death (Fig. 13). This was shown in some sample cases to be due to the respective hydrocarbons or metabolites. This was found to be independent of any carcinogenic effect. The presence of a palpable mass was also found to bear no relation to the presence of a tumour, often being due to the production of cysts filled with keratin.

TABLE III.—*Incidence of Tumours in a Series of Embryo Implants Exposed to Hydrocarbons*

| Hydrocarbon | Stomach | Lung | Skin | Bladder |
|---|---|---|---|---|
| Pyrene | 0/3 | 1A/1 | 0/3 | 0/1 |
| 1,2-Benzopyrene | 0/4 | 0/3 | 0/2 | 0/2 |
| 3,4-Benzopyrene | 1/3 | 2/2 | 2/2 | 0/1 |
| 1,2 : 3,4-Dibenzopyrene | 1/3 | 2/3 | 2/2 | 1/3 |
| 1,2 : 4,5-Dibenzopyrene | 0/1 | 1/2 | 1/5 | 0/4 |
| 1,2 : 6,7-Dibenzopyrene | 0/3 | 0/3 | 0/3 | 0/3 |
| 3,4 : 8,9-Dibenzopyrene | 0/1 | 1/1 | 1/1 | 0/1 |
| 3,4 : 9,10-Dibenzopyrene | 6/6 | 6/6 | 4/4 | 2/2 |
| Anthracene | 0/2 | 0/3 | 0/4 | 0/1 |
| 1,2-Benzanthracene | 0/2 | 3A/3 | 0/3 | 0/3 |
| 1,2 : 3,4-Dibenzanthracene | 0/2 | 3A/3 | 0/2 | 0/3 |
| 1,2 : 5,6-Dibenzanthracene | 2/2 | 2/3 | 0/3 | 2/3 |
| 1,2 : 7,8-Dibenzanthracene | 0/3 | 2/2 | 0/1 | — |

$\text{Ratio} = \frac{\text{Tumour induction}}{\text{Embryo implants}}$ A = Adenoma without carcinoma.

From Table III it is seen that tumours were induced in more than one type of tissue by each of the carcinogens, thus giving a wider scope than other methods. In some cases adenomas occurred in the embryo lung implants with or without accompanying squamous carcinomas (Fig. 14, 15). Where adenomas occurred their presence was recorded, but not put forward as proof of carcinogenicity on the part of the hydrocarbons to which the lung implant had been exposed. This course was decided upon because of the equivocal position of the spontaneous adenomas of lung in mice with regard to their classification as malignant lesions. Carcinomas were accepted as proof of carcinogenic action.

Some of the stomach implants showed an alteration in the connective tissue of the forestomach (Fig. 16, 17). There was a development of polypoidal processes and bridges covered by apparently normal squamous epithelium. Sometimes this was the only abnormal finding in the graft; in others it was found to be continuous with well developed squamous carcinoma. It is therefore felt that this appearance when present can be interpreted as pre-malignant and indicative of the need for a longer induction period than had been given up to the time of killing the particular animal.

SUMMARY

Having compared directly by a standard method 13 polycyclic aromatic hydrocarbons, the points of interest which emerge are as follows :

1. Blind testing of known substances gave results as reliable as those by previous methods, and correlated with what was already known of their carcinogenic properties.

2. The induction of tumours in more than one type of tissue by each of the known carcinogens was noted.

3. The short time of 16 weeks required to obtain the results.

4. The very small amounts of material required for each test (not more than 150 $\mu$g.).

5. The simplicity of this single step procedure.

It is felt therefore that this method fulfils the criteria postulated at the beginning of the paper and that it is a procedure which can safely be used for the bioassay of untested suspect substances.

This investigation was supported by a full time Research Grant from the British Empire Cancer Campaign.

REFERENCES

Fell, G. S. and Spence, J. B.—(1962) *VIIIth International Cancer Congress (Moscow) Abstracts of Papers*, p. 151.

Peacock, P. M. and Dick, Elizabeth—(1963) *Brit. J. Cancer*, in press.

Rous, P. and Smith, W. E.—(1945) *J. exp. Med.*, **81**, 597.